# Supplementary material for: Segment-specific optogenetic stimulation in Drosophila melanogaster with linear arrays of organic light-emitting diodes
Source: Nat Commun. 2020 Dec 7;11:6248. doi: 10.1038/s41467-020-20013-6 (PMC7721879; doi:10.1038/s41467-020-20013-6)
Supplement: Supplementary file 1 — Supplementary Information [file 41467_2020_20013_MOESM1_ESM.pdf]

# Supporting Information: Segment-Specific Optogenetic Stimulation in *Drosophila melanogaster* with Linear Arrays of Organic Light-Emitting Diodes


Caroline Murawski,[1,2] Stefan R. Pulver,[3] and Malte C. Gather[1,4]*

1 Organic Semiconductor Centre, SUPA, School of Physics and Astronomy, University of St Andrews, St Andrews KY16 9SS, UK

2 Kurt-Schwabe-Institut für Mess- und Sensortechnik Meinsberg e.V., Kurt-Schwabe-Str. 4, 04736 Waldheim, Germany

3 School of Psychology and Neuroscience, University of St Andrews, St Mary's Quad, South Street, St Andrews KY16 9JP, UK

4 Centre for Nanobiophotonics, Department Chemie, University of Cologne, Greinstr. 4-6, 50939 Köln, Germany

* Corresponding author: mcg6@st-andrews.ac.uk

## Supplementary Figures

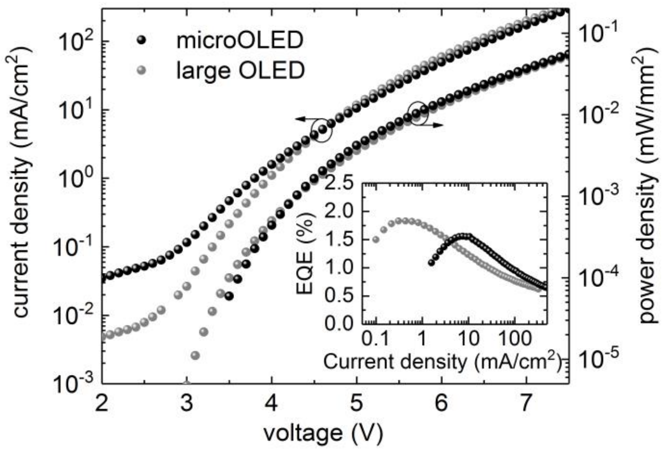


**Supplementary Figure 1.** Current density and optical power density as a function of the voltage for large (4 × 4 mm$^2$) and for microstructured OLEDs. Inset: External quantum efficiency as a function of the current density.

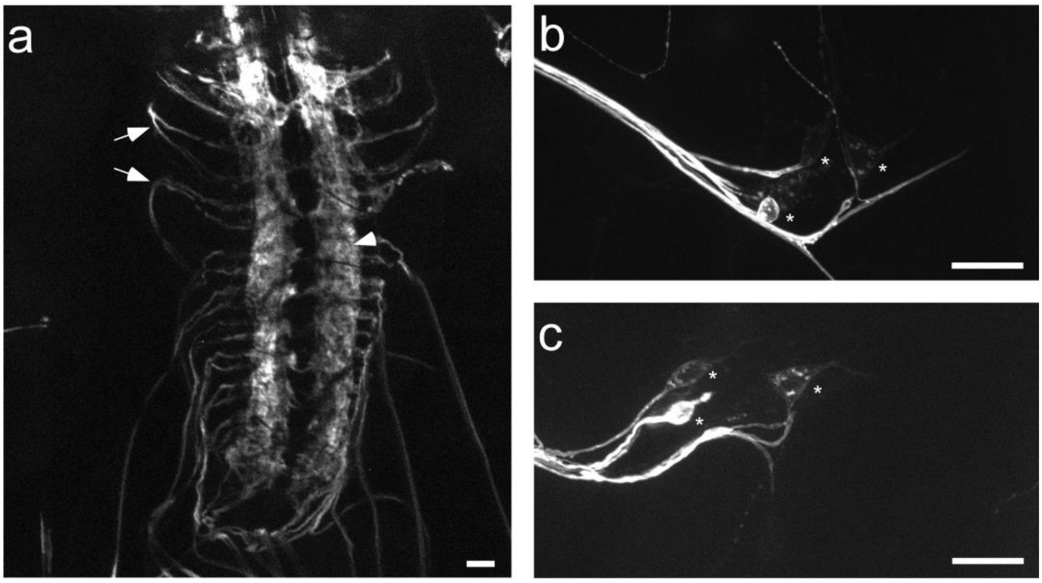


**Supplementary Figure 2.** Expression pattern of 5-40-GAL4. a) 3$^{rd}$ instar larval ventral nerve cord showing dense CsChrimson expression in neuropil regions (arrowhead) and nerve roots (arrows). Note lack of expression in any cell bodies in the central nervous system. b,c) 5-40-GAL4 expression in sensory neuron cell bodies in the peripheral nervous system (asterisks). Scale bars: 20 µm in all panels.

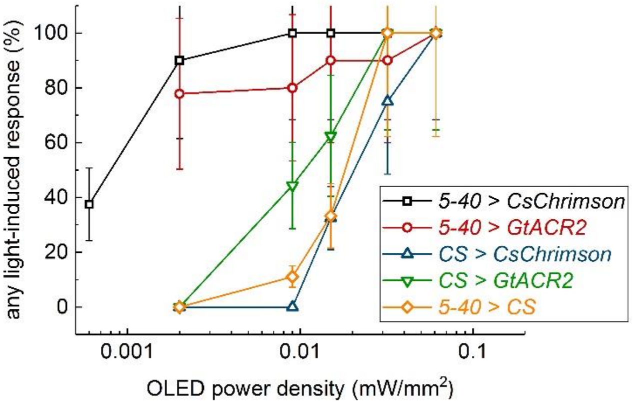


**Supplementary Figure 3.** Dose-response curve for detecting any light-induced behaviour, e.g., twitching or stronger head movements, during exposure to blue light illumination from 4×4 mm$^2$ OLEDs. Control larvae showed a light-evoked behaviour for intensities of around 15 µW mm$^{-2}$ and higher. Data shows mean ± SEM; *n* ≥ 7 larvae in each genotype.

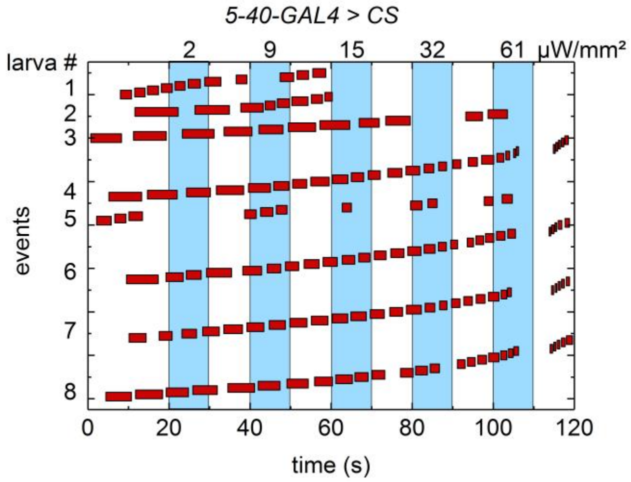


**Supplementary Figure 4.** Wave duration of forward muscle contraction waves upon stimulation with a large OLED at different brightness levels (OLED power density as indicated in top axis) for 5-40-GAL4 > CS larvae. Different events are spread across the y-axis. Numbers label events from individual animals (*n* = 8 larvae). Muscle contraction waves significantly accelerated at power densities above 32 µW mm$^{-2}$ and were accompanied with strong head and tail movements (Supporting Movie 3).

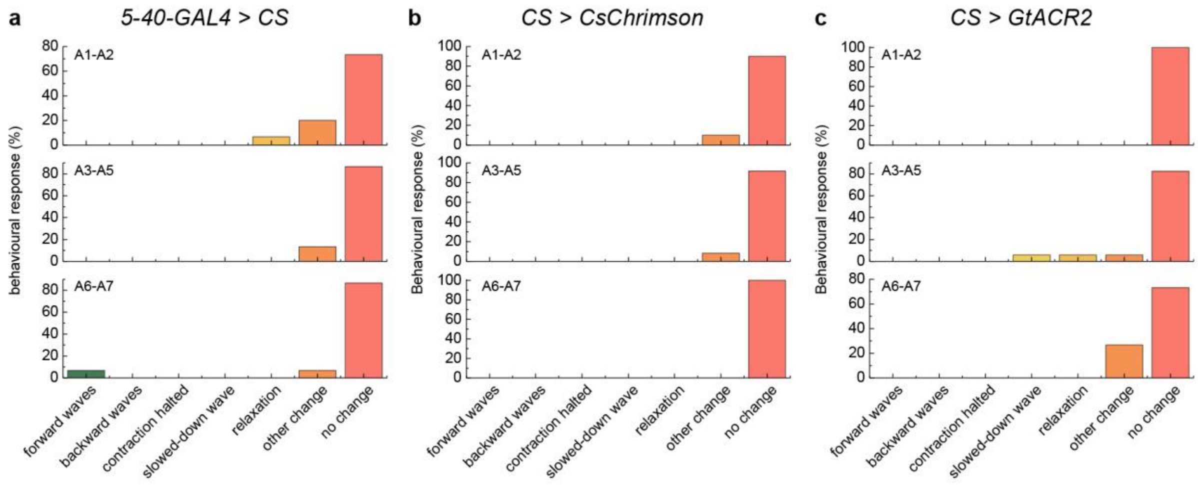


**Supplementary Figure 5.** Behavioural response of controls upon local stimulation with three neighbouring OLED pixels delivering 15 μW $mm^{-2}$ for 10 s. Most larvae (> 70 % for each condition) showed no response. Some larvae showed a change in behaviour, however, this is attributed to random, non-light-induced changes. Importantly, no muscle contraction waves were evoked. $n \geq 4$ larvae for each condition.
