# Supplementary material for: Segment-specific optogenetic stimulation in Drosophila melanogaster with linear arrays of organic light-emitting diodes
Source: Nat Commun. 2020 Dec 7;11:6248. doi: 10.1038/s41467-020-20013-6 (PMC7721879; doi:10.1038/s41467-020-20013-6)
Supplement: Supplementary file 3 — Description of Additional Supplementary Files [file 41467_2020_20013_MOESM3_ESM.pdf]

Title: Supplementary Movie 1.
Description: Whole animal stimulation during forward crawling in a GtACR2-expressing larva using a large OLED pixel. Upon stimulation, relaxation of the larva or slow-down of muscle contraction waves were recorded. The 10 s-long periods during which the OLED is on are indicated in the video. OLED power density: 15 µW mm-2.

Title: Supplementary Movie 2.
Description: Dose-response upon whole animal stimulation of a CsChrimson-expressing larva using a large OLED pixel. The 3 s-long periods during which the OLED is on, the brightness, and the larval response are indicated in the video.

Title: Supplementary Movie 3.
Description: Dose-response upon whole animal stimulation of a control larva using a large OLED pixel. The 10 s-long periods during which the OLED is on, the brightness, and the larval response are indicated in the video.

Title: Supplementary Movie 4.
Description: Local stimulation of a CsChrimson-expressing larva in segment A5 using a microstructured OLED leads to a temporary stop of an incoming forward muscle contraction wave. Bottom: Location of the muscle contraction wave over time. The 3 s- long period during which the OLED is on is indicated in the video. OLED power density: 15 µW mm-2.

Title: Supplementary Movie 5.
Description: Switching the direction of locomotion of a CsChrimson-expressing larva by alternating stimulation of anterior and posterior segments with a microstructured OLED. Red lines indicate the current position of the muscle contraction wave. The 3 s-long periods during which the OLED is on are indicated in the video. OLED power density: 15 µW mm-2.

Title: Supplementary Movie 6.
Description: Wave-like optical stimulation of 5-40-GAL4 > CsChrimson larvae from posterior to anterior by targeted local stimulation with a microstructured OLED. The location and periods during which the OLEDs were on are indicated in the video. OLED power density: 30 µW mm-2.
